# Lipid Profile Perturbations Associated With Subclinical Hypothyroidism: A Descriptive Study

**DOI:** 10.7759/cureus.58181

**Published:** 2024-04-13

**Authors:** Sara Harrar, Ibtissam Mhirig, Yazid El Alaoui Boufares, Ayoub Bouchehboun, Fatima Bounani, Siham Aboulmakarim

**Affiliations:** 1 Department of Clinical Biochemistry, Mohamed VI Training and Research Hospital, Faculty of Medicine and Pharmacy, Cadi Ayyad University, Marrakech, MAR; 2 Department of Clinical Biochemistry, Mohamed VI Training and Research Hospital, Faculty of Medicine and Pharmacy, Cadi Ayyad University, Marrakesh, MAR

**Keywords:** thyroid hormones, lipid profile, subclinical hypothyroidism, dyslipidemia, hypothyroidism

## Abstract

Background

Hypothyroidism is a prevalent endocrine disorder associated with dyslipidemia, which increases cardiovascular risk. Our study aimed to estimate the prevalence of dyslipidemia and subclinical hypothyroidism (SCH) and their correlation in a diverse population.

Methods

A descriptive cross-sectional retrospective analysis was conducted to assess the prevalence of dyslipidemia in patients with SCH. Data were collected over 19 months from the Clinical Biochemistry Department of a Moroccan university hospital. A total of 447 patients were included based on comprehensive lipid profile and thyroid-stimulating hormone (TSH) assessments, and normal free thyroxine (FT4) levels. Lipid profile and TSH measurements followed standardized procedures using the Cobas Roche® 6000 system (Roche Diagnostics Corporation, Indianapolis, USA). Dyslipidemia and SCH were defined according to established thresholds recommended by reputable organizations. Statistical analyses were performed using SPSS version 23.0 (IBM Corp., Armonk, USA) and Microsoft Excel (Microsoft Corporation, Redmond, USA), with significance set at p < 0.05*.*

Results

In the total population (447 individuals), the prevalence of dyslipidemia was approximately 42.05% (N = 188), with hypoHDLemia being most prevalent at approximately 31.31% (N = 140). The prevalence of SCH was approximately 12.75% (N = 57), with women constituting approximately 7.6% and men approximately 5.15%. In the euthyroid group 1 (N = 390), the prevalence of dyslipidemia was approximately 40.76% (159 individuals), while in the hypothyroid group 2 (N = 57), it increased to approximately 50.87% (N = 29). Hypertriglyceridemia was more prevalent in Group 2, with a prevalence of approximately 21.05% (N = 12), compared to Group 1, which had a prevalence of approximately 13.84% (N = 54). Additionally, hypoHDLemia was notably higher in Group 2, with a prevalence of approximately 38.59% (N = 22), compared to Group 1, which had a prevalence of approximately 30.25% ( N = 118).

The chi-square test revealed a significant association between SCH and dyslipidemia (χ2 = 1.427, p < 0.05). The calculated odds ratio (OR) of 1.5 (p < 0.05) indicates that individuals with SCH are 1.5 times more likely to have dyslipidemia compared to those without SCH.

Conclusion

In conclusion, our study provides valuable insights into the prevalence of dyslipidemia and its association with SCH in our patient population. We observed a notable prevalence of dyslipidemia among individuals with SCH, characterized by elevated levels of total cholesterol (TC) and low-density lipoprotein cholesterol (LDL-C). Importantly, while chi-square tests revealed a significant association between SCH and dyslipidemia, logistic regression analyses did not confirm a statistically significant correlation after adjusting for potential confounders.

## Introduction

Hypothyroidism, a common endocrine disorder, results from an insufficient production of thyroid hormones by the thyroid gland. Thyroid hormones, primarily thyroxine (T4) and triiodothyronine (T3) play a pivotal role in regulating metabolic processes, energy balance, and overall homeostasis within the body [1-2]. The deficit of these hormones in hypothyroidism can lead to a wide range of clinical manifestations, making it a condition of significant medical concern [3].

Hypothyroidism is a global health issue, affecting millions of individuals worldwide. Its prevalence varies across populations and age groups, with a predilection for females and an increased incidence with advancing age [4]. The condition presents a considerable public health challenge, warranting comprehensive research and clinical management strategies.

One of the notable complications associated with hypothyroidism is dyslipidemia, characterized by disturbances in blood lipid profiles. Dyslipidemia is a recognized risk factor for cardiovascular diseases (CVD), including atherosclerosis, coronary artery disease, and stroke [5-6]. In the context of hypothyroidism, patients frequently exhibit alterations in lipid metabolism, typically characterized by elevated levels of total cholesterol (TC), low-density lipoprotein cholesterol (LDL-C), and triglycerides (TG), along with reduced high-density lipoprotein cholesterol (HDL-C) [7-8]. The dyslipidemia observed in hypothyroid patients can further exacerbate their cardiovascular risk, necessitating close attention to lipid management in this population [9].

While there is a wealth of global research that underscores the connection between hypothyroidism and dyslipidemia, the significance of localized data cannot be overstated. This study aims to address this need by confirming the prevalence of dyslipidemia among subclinical hypothyroid patients in our region, to enhance the prevention and therapeutic management of this condition.

## Materials and methods

Study design and protocol

This descriptive cross-sectional retrospective study aimed to investigate the prevalence of dyslipidemia in patients with subclinical hypothyroidism (SCH). Data were collected from the Clinical Biochemistry Department of the Mohamed VI University Hospital in Marrakech, which is the only tertiary hospital in the region that receives patients referred from the south of Morocco. The study spanned a period of 19 months, from December 2019 to July 2021.

Sampling strategy - inclusion and exclusion criteria

A total of 447 patients were considered for inclusion in the study. These patients were drawn from the patient population at Mohamed VI University Hospital. The inclusion criteria encompassed all patients from various clinical departments who had undergone a comprehensive lipid profile assessment, including measurements of TC, TG, HDL-C, LDL-C, and thyroid-stimulating hormone (TSH) measurement, and had a normal dosage of free thyroxine (FT4) and T3.

Exclusion criteria were applied to individuals with incomplete assessments or those presenting abnormally low T3 and FT4 levels.

All the individuals included in the study had no past medical history of hypothyroidism and were not under treatment for hypothyroidism

Random sampling

The sampling approach utilized in this study involved random sampling, whereby data were collected from every eligible patient who met the inclusion criteria from historical records. This random sampling method enhances the representativeness of the samples and reduces selection bias.

Data collection

Retrospective Data Collection: Data were collected from historical laboratory records. These records included patients who met the inclusion criteria.

Sample collection and analysis

The patient’s age, gender, and the prescribing department were recorded, and approximately 5 ml of venous blood was collected in the morning following a 12-hour fast using vacutainer tubes with a gel separator. The samples were then centrifuged at 2000 revolutions per minute for 10 minutes.

Standardized procedures

Sample collection, handling, and storage were performed according to standardized procedures to ensure sample integrity. Lipid profile and TSH measurements followed internationally recognized guidelines and protocols for accurate and consistent results

Assay methods

All parameter measurements were performed using the Cobas Roche® 6000 automated system (Roche Diagnostics Corporation, Indianapolis, USA). TSH measurement was conducted through a two-step chemiluminescence immunoassay (CMIA). HDL, TG, and TC levels were determined using enzymatic colorimetric methods. LDL-C (calculated LDL) was calculated using the Friedewald formula: LDL = total cholesterol - (HDL cholesterol - triglycerides) / 5."

Quality control

Quality control measures were implemented to maintain the accuracy and reliability of the data. Regular calibrations and daily controls for laboratory equipment were conducted. Lipid profile and TSH measurements adhered to well-established international guidelines and protocols.

Data completeness

Data completeness was ensured by verifying that the collected data were complete and that there were no missing values.

Definition of thresholds

TSH Threshold

For the definition of subclinical hypothyroidism (SCH) in this study, a TSH threshold of 4.0 µIU/mL, and a normal dosage of FT4 level within the range of 0.6 to 1.6 ng/dL, were adopted. Therefore, patients with a serum TSH level exceeding this threshold were classified as cases of subclinical hypothyroidism. This threshold aligns with the recommendations of reputable organizations, including the American Thyroid Association (ATA) [10] and the Endocrine Society [11]. Both the ATA and the Endocrine Society suggest a TSH reference range between 0.4-4.0 µIU/mL for the diagnosis of thyroid disorders.

Dyslipidemia Thresholds 

For this study, dyslipidemia was defined based on threshold values recommended by the American Heart Association (AHA) [12] and the National Cholesterol Education Program (NCEP) guidelines [13]. The thresholds used to classify individuals with dyslipidemia are as follows:

Total Cholesterol (TC): Dyslipidemia is defined as TC levels equal to or greater than 240 mg/dL, which corresponds to the "high" value according to the guidelines [12]

Triglycerides (TG): Dyslipidemia is defined as TG levels equal to or greater than 200 mg/dL, corresponding to the "high" value as per the guidelines [12]

HDL Cholesterol (HDL-C): Dyslipidemia is defined as HDL-C levels less than 40 mg/dL for men and less than 50 mg/dL for women, both of which represent the lower limits for what is considered normal [12]

LDL Cholesterol (LDL-C): Dyslipidemia is defined as LDL-C levels equal to or greater than 160 mg/dL, corresponding to the "high" value as per the guidelines [13]

Statistical analysis

All statistical analyses were performed using the Statistical Package for the Social Sciences (SPSS) version 23.0 (IBM SPSS Statistics, Armonk, USA) and Microsoft Excel (Microsoft Corporation, Redmond, USA). Categorical data were expressed as percentages (%) and compared with the χ2 test. Measurement data of normal distribution were expressed as mean ± SD and a t-test was used for data comparison. P < 0.05 indicates that the difference is statistically significant.

## Results

Total population

Demographics

**Figure 1:**
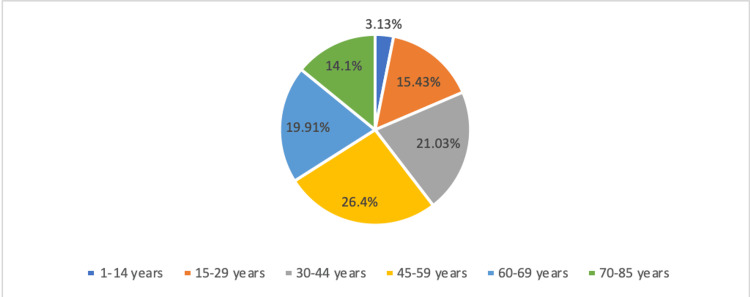
Distribution of the studied population by age groups

Total number of patients: 447, composed of 62,6% of women (n=280) and 37.36 % of men (n=167); the sex ratio male/female was 0.596. The mean age was 48.84 years ± 17.677, with a range age from 1 year to 85 years old. (Figure 1)

Descriptive Statistics

The mean TSH value was 3.78 µIU/mL, with a standard deviation of 1.619. The mean lipid profile levels were - total cholesterol 177mg/dl, triglycerides 135 mg/dl, LDL 102 mg/dl, HDL 48 mg/dl, with corresponding standard deviations of: 0.332, 0.296, 0.275, 0.113.

Prevalence Analysis

The overall prevalence of SCH in the total population was 12.75% (95% confidence interval (CI) 9.68-15.82), with women accounting for 7.6% and men for 5.15% of this total. The prevalence of SCH among women was 12.14 % (95% CI 7.53-16.75), versus 13.77 % among men (95% CI 8.79-21.45). The overall prevalence of dyslipidemia in the total population was 42.05% (95% CI 36.82-47.28). Hypercholesterolemia was present among 8.5 % of the total population ( accounting for 5.59 % of women and 2.9% of men). Hypertriglyceridemia was present among 14.76 % of the total population ( accounting for 9.17 % of women and 5.59% of men). HyperLDLemia was present among 7.82 % of the total population ( accounting for 4.25 % of women and 3.57% of men). And hypoHDLemia was present among 31.31% of the total population, with the same distribution in men and women (15.65%)

The prevalence of dyslipidemia among women constituted 37.85%, corresponding to 23.71 % of the total population. 25% of women presented HypoHDLemia, 14.64% hypertriglyceridemia, 8.9% hypercholesterolemia, and 6.78 % hyperLDLemia. The prevalence of dyslipidemia among men constituted 49.1%, corresponding to 18.34% of the total population. 41.91% of men presented HypoHDLemia, hypertriglyceridemia was present among 14.97% of men, hypercholesterolemia among 7.78%, and hyperLDLemia among 9.58% of them.

Age-Stratified Analysis

The key observations from our examination of TSH levels and lipid profiles across different age groups showed that mean TSH values exhibited a distinct pattern, with the highest levels observed in the 45-59 years age group (5.66), followed by the 15-29 years age group, and the lowest in the 1-14 years age group (1.98). Lipid parameters displayed a relatively consistent profile across these age segments, indicating a degree of stability in lipid metabolism, as shown in Table 1

**Table 1:** Mean values of TSH and lipid profile by age group mIU/L: milli-international units per liter: LDL: low-density lipoprotein; HDL: high-density lipoprotein; mg/dL: milligram/deciliter; TC: total cholesterol; TG: triglyceride; TSH: thyroid-stimulating hormone.

Mean value	1-14 years	15-29 years	30-44 years	45-59 years	60-69 years	70-85 years
TSH (mIU/L)	1.98	4.54	2.98	5.66	2.67	2.47
Total Cholesterol (mg/dL )	143	184	182	190	175	149
Triglycerid (mg/dL)	135	129	138	138	137	126
LDL (mg/dL)	75	109	103	111	104	79
HDL (mg/dL)	40	51	50	48	47	45

**Figure 2:**
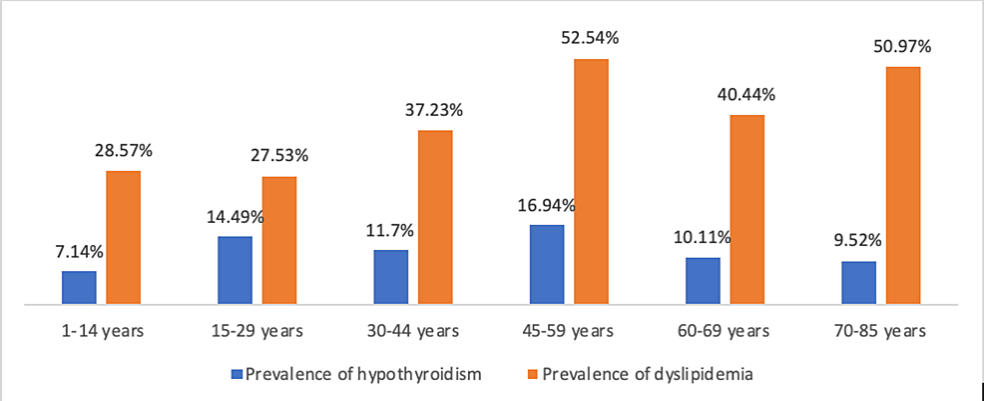
Prevalence of SCH and dyslipidemia among age groups SCH: subclinical hypothyroidism

As we transition from the age-stratified analysis to a more detailed exploration of euthyroid and hypothyroid groups, it is essential to highlight that the age group 45-59 years showed the highest prevalence of SCH (16.94%) and dyslipidemia (52.54%) as shown in Figure 2. The demographics and main values among age groups are shown in Table 2.

**Table 2:** Demographics and main values among age groups TSH: thyroid-stimulating hormone (mIU/L); TC: Total cholesterol (mg/dL); TG: triglyceride (mg/dL); LDL: low-density lipoprotein (mg/dL); HDL: high-density lipoprotein (mg/dL)

Age groups	1-14 (N=14)		15-29 (N=69)		30-44 (N=94)		45-59 (N=118)		60-69 (N=89)		70-85 (N=63)	
Sex groups	Male	Female	Male	Female	Male	Female	Male	Female	Male	Female	Male	Female
Demographics	8	6	20	49	30	64	40	78	38	51	31	32
Mean TSH	1.4	2.74	5.38	4.19	1.91	3.48	6.49	5.23	2.88	2.67	2.57	2.37
Mean TC	139	148	192	181	175	185	195	187	154	190	140	158
Mean TG	103	178	153	119	143	135	147	132	130	143	123	129
Mean LDL	77	72	128	101	102	103	122	106	93	112	74	83
Mean HDL	41	39	40	56	43	53	44	50	41	52	42	49

These findings lay the groundwork for a more focused examination of euthyroid and hypothyroid individuals in Groups 1 and 2, shedding light on potential correlations between thyroid function and lipid profiles.

Euthyroid group (Group 1)

Demographics

The group of euthyroid patients was composed of a total of 390, with 246 women and 144 men, a sex ratio of 0.58, and a mean age of 48.92 ± 17.67.

Descriptive Statistics

The mean TSH value was 1.69 µIU/mL, with a standard deviation of 1.619. The mean lipid profile levels were - total cholesterol 176 mg/dl, triglycerides 134 mg/dl, LDL 101 mg/dl, HDL 48 mg/dl, with corresponding standard deviations: 0.332, 0.296, 0.275, 0.113.

Dyslipidemia Analysis

The overall prevalence of dyslipidemia in the euthyroid group was 40.76%. Hypercholesterolemia was present among 8.2%, hypertriglyceridemia was present among 13.84% of Group 1, hyperLDLemia was present among 7.43%, and hypoHDLemia was present among 30.25% of the euthyroid group.

The prevalence of dyslipidemia among women in the euthyroid group was 35.36%. Among women of the euthyroid group, 27.64% had hypoHDLemia, 13.82% had hypertriglyceridemia, 8.94% had hypercholesterolemia, and 7.31% had hyperLDLemia. The prevalence of dyslipidemia among men composing the Group 1 constituted 50%. Among men of Group 1, 34.72% of men presented hypoHDLemia, hypertriglyceridemia was present among 13.88% of men, hypercholesterolemia among 6.94%, and hyperLDLemia among 7.63% of them.

Hypothyroid group (Group 2)

Demographics

The group of hypothyroid patients was composed of a total of 57, with 34 women and 23 men, a sex ratio of 0.67, and a median of 51 (37, 60)

Descriptive Statistics

The median TSH value was 7.52 µIU/mL (5.45, 14.62). The mean lipid profile levels were - total cholesterol 185 mg/dl, triglycerides 139 mg/dl, LDL 111 mg/dl, HDL 48 mg/dl, with corresponding standard deviations of: 0.219, 0.544, 0.113, and 0.007, respectively.

Dyslipidemia Analysis

The overall prevalence of dyslipidemia in the hypothyroid group was 50.87%. Hypercholesterolemia was present among 10.52% (5.26% of women and 5.26% of men). Hypertriglyceridemia was present among 21.05% of Group 2 (composed of 15.78% women and 5.26% of men). HyperLDLemia was present among 10.52% (equally reported between men and women). And hypoHDLemia was present among 38.59% of the hypothyroid group (22.8% women and 15.78% men).

The prevalence of dyslipidemia among women in the hypothyroid group was 55.88%. Among women of Group 2, 38.23% presented HypoHDLemia, 26.47% hypertriglyceridemia, 8.82% hypercholesterolemia, and 8.82 hyperLDLemia. The prevalence of dyslipidemia among men composing Group 2 constituted 43.47%. Among men of Group 2, 39.13% presented hypoHDLemia, and hypertriglyceridemia, hypercholesterolemia, and hyperLDLemia were present equally with a percentage of 13.04.

Statistical analysis 

Pearson Correlation Coefficients

The Pearson correlation coefficient was employed to assess the strength and direction of the linear relationship between TSH levels and lipid parameters across the three groups. In the total population, our analysis revealed weak associations: total cholesterol (r = 0.073), triglycerides (r = 0.0563), HDL (r = -0.029), and LDL (r = 0.07). Group 1, representing euthyroid individuals, exhibited slightly stronger correlations: total cholesterol (r = 0.125), triglycerides (r = 0.0979), HDL (r = 0.0919), and LDL (r = 0.1001). However, it was in Group 2, the hypothyroid group, that the correlations were notably more pronounced: total cholesterol (r = 0.126), triglycerides (r = 0.1604), HDL (r = -0.0829), and LDL (r = 0.1205).

T-Test

To further investigate the impact of TSH levels on lipid profile, a t-test was conducted to compare means of euthyroid (Group 1) and hypothyroid (Group 2) patients (Table 3).

**Table 3:** Comparison of lipid parameters in participants of group 1 and 2 LDL: low-density lipoprotein; HDL: high-density lipoprotein; mg/dL: milligram/deciliter; SCH: subclinical hypothyroidism; TC: total cholesterol; TG: triglyceride.

Lipid parameters (mg/dL)	Group 1 Euthyroid (n=390)	Group 2 SCH (n=57)	p-value
TC	176 ± 0.332	185 ± 0.219	0.1364
TG	134 ± 0.296	139 ± 0.544	0.3962
LDL	101 ± 0.275	111 ± 0.113	0.1754
HDL	48 ± 0.113	48 ± 0.007	1.0000

The results of the t-test indicated no significant difference in lipid profile parameters between euthyroid and hypothyroid individuals.

Chi-square Test

The chi-square test investigating the association between subclinical hypothyroidism and lipid abnormalities produced a significant result (χ2 = 1.427, p < 0.05). It revealed interesting findings when comparing the prevalence of dyslipidemia between Group 1 (euthyroid individuals) and Group 2 (hypothyroid patients), especially when comparing females among the two groups ((χ2≈28.95)

In Group 1, the prevalence of dyslipidemia was 40.76%, while in Group 2, it increased to 50.87%. Notably, hypertriglyceridemia was more prevalent in Group 2 (21.05%) than in Group 1 (13.84%). Additionally, hypoHDLemia, a significant lipid profile alteration, was notably higher in Group 2 (38.59%) compared to Group 1 (30.25%). *Odds Ratio* The calculated odds ratio (OR) for the association between SCH and dyslipidemia in our study is 1.5 (0.86-2.62, p < 0.05)

## Discussion

​​​​​​Thyroid hormones (THs) play a crucial role in modulating various metabolic pathways, including basal energy expenditure and the metabolism of carbohydrates, proteins, and lipids [14]. Notably, thyroid disorders, encompassing overt and subclinical hypothyroidism (SCH), exert significant effects on lipid profiles, contributing to cardiovascular disease (CVD) [15]. THs are involved in regulating lipid synthesis, metabolism, and mobilization, leading to alterations in the composition and transport of lipoproteins (LPs)[16]. Studies consistently report elevated serum levels of total cholesterol (TC), LDL-C, apolipoprotein B, lipoprotein (a), and potentially triglycerides (TGs) in individuals with hypothyroidism [17-18]. Increased TG levels are associated with proatherogenic changes, including reduced cardioprotective high-density lipoprotein (HDL) and the generation of small dense LDL (sdLDL) [19-20].

Our study encompassed a diverse cohort of 447 individuals, there were more women than men in our study (62.64% vs 37.36%). This characteristic is similar to a study conducted in Colorado on 25,862 individuals, where there were 55.8% of women and 44.2 % of men. The mean age was slightly higher in this study (56.0) [4]

Delving into the descriptive statistics, we noted a mean TSH value of 3.78 µIU/mL. It was higher than the mean TSH value found in a study conducted by Iqbal et al [21]. Lipid profile measurements unveiled mean values of total cholesterol (177 mg/dl), triglycerides (135 mg/dl), LDL (102 mg/dl), and HDL (48 mg/dl). These findings correspond to a study conducted in the same country [22]

Moving to dyslipidemia, we observed an overall prevalence of 42.05% among the total population. A study conducted in the same country by Hsai et al. showed a higher prevalence (91.78%) [22]. The overall prevalence of dyslipidemia was higher in men (49.1%) compared to women (37.85%) among the total population in both studies [22].

Concerning the age-stratified analysis, Individuals within the age group 45-59 years were more likely to have SCH (16.94%), and dyslipidemia (52.54%). In the study conducted in Colorado, it was the individuals older than 74 years who were more likely to have SCH [4], in the study conducted by Yan Xie et al. [23] it was individuals older than 60 years (21.6%).

Our investigation into the prevalence of SCH revealed an overall rate of 12.75%. When compared to the detected rate in a cohort composed of 28 568 healthy individuals (11.10%) (95% CI 10.72-11.47), our observed prevalence appears slightly elevated [23]. It was also elevated compared to the cohort study conducted in Colorado (9.5%) [4]. The percentage of subjects with an elevated TSH concentration was greater for women than men (7.6% vs 5.15%). This result is similar to the study conducted in Colorado [4]

The cohort study reported a prevalence of SCH among women at 13.54%, surpassing the prevalence in men, which stood at 9.95% [23]. In contrast, our study identified a slightly lower prevalence in women, with 12.14%, and a marginally higher prevalence in men at 13.77%. These variations underscore the inherent complexity and multifactorial nature of thyroid disorders, influenced by diverse demographic and environmental factors.

Transitioning to a detailed exploration of euthyroid (Group 1) and hypothyroid (Group 2) individuals, the euthyroid group (Group 1) showed a mean TSH value of 1.69 µIU/mL ± 1.619. This result is similar to the study conducted by A. Iqbal et al. (TSH = 1.8) [21], and lower to the study conducted by M. Ejaz et al (TSH=3.12 ± 0.56) [24]. Group 2 showed a mean TSH value of 18.06 µIU/mL ± 59.75, this mean is higher compared to other studies showing means of 5.7 [21] and 6.58 [24]. 

Concerning the means of lipid profile, we note lower means in TC, LDL, and HDL, and higher in TG among Groups 1 and 2 compared to other studies [21-25]. The results of the t-tests between euthyroid and SCH indicated no significant difference in lipid profile parameters. In other studies, there was a significant correlation only for TC and LDL [4-21].

The prevalence of dyslipidemia was higher in Group 2 (50.87%) than in Group 1 (40.76%). This prevalence was higher in a study conducted in South Africa (70%) [26]. This difference could be attributed to several factors, including variations in dietary habits, sociodemographic characteristics, and genetic predispositions among the studied populations

Gender-specific analysis revealed variations, with dyslipidemia affecting women (55.88%) more than men (43.47%) in Group 2, and women (35.36%) less than men (50%) in Group 1. HypoHDLemia was the highest lipid abnormality among the two groups. But we note a higher prevalence of hypertriglyceridemia among individuals of Group 2 (21.05% vs 13.84% in Group 1), especially among women (26.47% vs 13.8% in Group 1).

We employed Pearson correlation coefficients to assess the relationship between TSH levels and lipid parameters across distinct groups. In the total population, the coefficients indicated weak associations, suggesting a mild connection between TSH and lipid profiles. However, in Group 2, while correlations were slightly stronger compared to Group 1, they still showed a relatively weak association.

The chi-square test was employed to explore the association between SCH and dyslipidemia. While the correlation coefficient and t-test results did not indicate significant associations, the chi-square test yielded a statistically significant result (χ2 = 1.427, p < 0.05), suggesting that the prevalence of dyslipidemia differs between euthyroid and hypothyroid groups. Although the correlation between TSH levels and lipid parameters was weak, the higher prevalence of dyslipidemia in the hypothyroid group underscores the potential influence of SCH on lipid profile alterations. While further investigation is warranted to elucidate the exact mechanisms underlying this association, our findings emphasize the clinical significance of monitoring lipid parameters in hypothyroid patients.

Based on the conducted chi-square test, a significant association between SCH and lipid abnormalities was observed (χ2 = 1.427, p < 0.05). Regarding the odds ratio (OR) analysis, the calculated odds ratio for the association between SCH and dyslipidemia was found to be 1.5 (0.86-2.62, p < 0.05) The logistic regression analysis further elucidated the role of demographic factors. While age did not exhibit a statistically significant association with dyslipidemia (p = 0.258), gender showed a significant association (p = 0.013). Specifically, females were found to have 1.643 times higher odds of dyslipidemia compared to males. However, the status of hypothyroidism did not show a significant influence on the risk of dyslipidemia (p = 0.248) after adjusting for confounding factors. This difference between the chi-square test and the logistic regression can be explained by the imbalance in the distribution of genders in the sample. The absence of an association may be because the majority of subjects with hypothyroidism were male, while there were more females in the sample.

## Conclusions

In conclusion, our study provides insights into the prevalence of dyslipidemia and its association with subclinical hypothyroidism (SCH) in our patient population. We observed a notable prevalence of dyslipidemia characterized by elevated levels of total cholesterol (TC) and low-density lipoprotein cholesterol (LDL-C). While chi-square tests revealed a significant association between SCH and dyslipidemia, logistic regression analyses did not confirm a statistically significant correlation after adjusting for potential confounders.

These findings suggest that while there is a noteworthy co-occurrence of SCH and dyslipidemia, other factors beyond thyroid dysfunction may contribute to the development of abnormal lipid profiles. Therefore, future research should explore additional variables such as lifestyle factors, genetic predisposition, and comorbidities to better understand the complex interplay between thyroid function and lipid metabolism.
